# Extracellular vesicles as the “magic bullet” for fighting threats to humanity

**DOI:** 10.20517/evcna.2021.14

**Published:** 2021-09-18

**Authors:** Takaaki Tamura, Yusuke Yoshioka, Takahiro Ochiya

**Affiliations:** ^1^Department of Molecular and Cellular Medicine, Institute of Medical Science, Tokyo Medical University, Tokyo 160-0023, Japan.; ^2^Department of Urology, Graduate School of Medicine, Chiba University, Chiba 260-8670, Japan.

**Keywords:** Extracellular vesicles, therapeutic exosomes, drug delivery system, COVID-19

## Abstract

Many researchers worldwide are currently trying to develop targeted molecular therapies such as nucleic acid medicines or antibody-drug conjugates for various diseases. Writing in *Extracellular Vesicles and Circulating Nucleic Acids*, Kim *et al. *summarized existing technologies for encapsulating therapeutic molecules into exosomes and introduced some human cell lines which are able to produce safe, effective therapeutic exosomes. Their review article offers the “magic bullet” for fighting threats to humanity such as the current coronavirus pandemic.

The “magic bullet” is a famous scientific idea advocated by Dr. Paul Ehrlich around 1900^[1]^. He was a German Nobel laureate, and he suggested that it could be possible to attack specific microbes which induce diseases without harming the body itself. He named this ideal agent a “magic bullet”. Dr. Ehrlich’s discovery in 1909 of arsphenamine for the treatment of syphilis is considered to be the first magic bullet. This finding led to the concept of therapeutic strategies for various diseases, including therapies against infections and chemotherapy. Many researchers worldwide are currently trying to develop targeted molecular therapies such as nucleic acid medicine or antibody-drug conjugates. However, the therapeutic effects of these treatments are still limited because it is difficult to deliver sufficient doses of a drug to a local lesion. For clinical application, it is necessary to develop innovative drug delivery systems (DDSs) that can efficiently deliver drug molecules to a lesion. As Dr. Ehrlich pointed out, the materials used for this new delivery system need to be safe for our bodies.

Emerging evidence suggests that extracellular vesicles (EVs), such as exosomes, can be promising candidate materials for DDS. It was discovered in the mid-2000s that EVs containing miRNAs and mRNAs circulate in our bodies^[2,3]^. After this discovery, around 2010, it was shown that miRNAs encapsulated in EVs function in recipient cells^[4-6]^. In 2015, Zomer *et al.*^[7]^ visualized intercellular molecular exchanges between tumor cells *in vivo* and showed that this phenomenon affects cellular metastatic behavior. These discoveries indicated that EVs play an active role in intercellular communication by transferring cellular materials to recipient cells, and they offer great potential as natural therapeutic delivery vehicles. Hoshino *et al.*^[8] ^demonstrated that molecules present on tumor-derived exosomes allowed them to target specific organs. This directivity is considered to extend the possible uses of exosome-based magic bullets.

Dr. Chois’ team is one of the leading research groups investigating exosome-based DDS and has made several contributions to this field. “EXPLOR”, an optogenetically engineered exosome system capable of loading adequate doses of therapeutic proteins into exosomes, was developed by his group^[9]^. This review paper introduced some existing technologies for encapsulating therapeutic molecules into exosomes and discussed some human cell lines that produce exosomes, as promising materials for preparing safe therapeutic exosomes. They also discussed the use of naïve exosomes which produce their own therapeutic effects. It is desirable to use normal human cells or cell lines for preparing safe therapeutic agents, but there are several problems which still need to be considered. As Kim *et al.*^[10]^ described in this review paper, scalability, consistency, and controllable manufacturing methods for culture will need to be established. Other sources of EVs have also been studied^[11-14]^. Some researchers previously studied the biological activities of bovine milk derived EVs (mEVs)^[11]^, and they have reported novel methods for applying mEVs to drug delivery vehicles^[12,13]^. In these studies, they demonstrated that no adverse effect was observed after serial administration of mEVs in mice, and they found that bovine milk could be a scalable source of EVs for mass production^[12,13]^. Other researchers examined the feasibility of orally administered nucleic acid drug delivery by acerola exosome-like nanoparticles^[14]^. However, in terms of exosome engineering, human cells or cell lines would be the best source, because we can easily manipulate specific regulatory genes or proteins in exosome producing cells. The review by Kim *et al.*^[10]^ is worthy of attention because the authors discuss these prospects and challenges for the clinical applications of exosome-based therapy in a straightforward manner.

To address the coronavirus pandemic, researchers worldwide are working on therapeutic approaches to COVID-19 using exosomes. Sengupta *et al.*^[15]^ have conducted a prospective, nonrandomized, open-label cohort study, and have evaluated the safety and efficacy of commercially available exosomes (ExoFlo™) derived from allogeneic bone marrow mesenchymal stromal cells (BM-MSCs) as a treatment for severe COVID-19. The treatment resulted in significant improvement in absolute neutrophil count and lymphopenia, with a decline in C-reactive protein, ferritin, and D-dimers. This is one of the first published clinical studies to use BM-MSC-derived exosomes as a treatment for COVID-19^[15]^. Some researchers believe that RNA interference can serve as a genetic treatment approach for critically ill individuals with SARS-CoV-2. Owing to their natural characteristics, exosomes are considered to be suitable carriers for the delivery of interfering RNA. There have been attempts to produce MSC-derived exosomes carrying a cocktail of the RNAs which inhibit targets involved in SARS-CoV-2 pathogenesis for the treatment of COVID-19 patients^[16]^.

Exosome-based therapies could be used in many types of diseases, including oncology and infectious diseases. The “EXPLORE” technology is innovative, and this kind of remarkable technology is based on deep insights into the biogenesis and delivery of exosomes. However, further insights and compliance with rigorous research and development guidelines are needed for clinical application. Recently, several significant position papers in this field have been provided to prevent any unproven EV therapies^[17,18]^. EV researchers need to follow the right path referring to these guide maps to overcome the immeasurable threats to humanity. These novel findings pave the way for future medical research, and we look forward to further development in this promising field.

## DECLARATIONS

### Copyright

© The Author(s) 2021.
